# Records of shallow landslides triggered by extreme rainfall in July 2024 in Zixing, China

**DOI:** 10.1038/s41597-025-05670-w

**Published:** 2025-08-05

**Authors:** Zijin Fu, Fawu Wang, Hao Ma, Qi You, Youqian Feng

**Affiliations:** 1 College of Civil Engineering, Tongji University, Shanghai, 200092 China; 2 Key Laboratory of Geotechnical and Underground Engineering, Ministry of Education, Tongji University, Shanghai, 200092 China

**Keywords:** Natural hazards, Environmental sciences

## Abstract

Global climate change has led to the frequent extreme meteorological events in recent years, triggering severe clustered landslides in mountainous regions. Records of these clustered landslides not only provide post-disaster statistics but also play a crucial role in advancing data-driven regional landslide research and intelligent landslide detection. The Rainfall-induced Landslide in Zixing (RLZX) datasets consist of a landslide inventory map (LIM) and a landslide detection dataset (LDD). RLZX-LIM was created through visual interpretation of 3D scenes before and after the rainfall event, containing 19,403 shallow landslides triggered by extreme rainfall in Zixing City, China, between July 26 and July 28, 2024. We have provided quantitative evaluations of the quality of RLZX-LIM based on reference data obtained from road-aligned surveys and unmanned aerial vehicle (UAV) mapping in the field. RLZX-LDD is further developed using both UAV and satellite images, offering higher quality and robustness, effectively filling the gap in rainfall-induced LDDs. The RLZX datasets have been publicly released for free use to promote related landslide research.

## Background & Summary

Landslides are geological processes in which rock and soil mass on a slope lose stability, fail, and move downward. These processes are widely developed in mountainous regions around the world, posing significant threats to infrastructure and human lives^1,2^. Earthquakes and rainfall events are the main external factors that trigger landslides in areas with complex terrain and fragile geological settings^3–5^. With the trend of global climate change, extreme rainfall events have become increasingly frequent, triggering numerous landslides in various regions^6^. Factors such as surface erosion, the reduction of geological material strength, and changes in pore pressure contribute to the rapid instability of initial slopes over a short period^7,8^. This instability, combined with flooding, creates significant destructive potential for landslides. The systematic recording of disasters triggered by such events is vital for advancing future disaster prevention and mitigation efforts.

Landslide inventory mapping is a fundamental task in regional landslide studies. A comprehensive landslide inventory map (LIM) should include records of the occurrence time, morphology, triggering factors, lithology, movement type, and the topographic and geological characteristics^9^. With the advancement of geographic information system (GIS) platforms, the recording of LIMs has become more convenient, with georeferenced points and polygons being the most commonly adopted formats for representing landslides^10^. Landslide polygons capture more detailed characteristics, while landslide points are often used for simplified modeling or on low-resolution available information^11–13^. Records of landslides occurring at different times and locations under various triggering factors can help researchers explore the temporal and spatial development characteristics of landslides as well as their environmental response patterns^14–17^. The LIMs provide a data-driven foundation for regional landslide research, encompassing susceptibility, hazard and risk^18–23^, which aim to predict the occurrence of landslides and assess their hazard and risk levels. Beyond fulfilling scientific research objectives, LIM is also important for providing valuable support for emergency management and post-disaster reconstruction.

The methods of building a LIM can be primarily classified into the following categories: field surveys, remotely sensed interpretation by experts, and automated or semi-automated methods^24,25^. With the advancement and widespread adoption of remote sensing techniques, an increasing amount of high-coverage, high-resolution and multi-temporal data, including optical^26,27^, multispectral^28^ and SAR images^29,30^ from satellites, UAV photographs^31^, and high-resolution DEMs^32^, are now available for LIM builders. Experts can annotate landslide boundaries by visual interpretation or choose the automated or semi-automated ways using these data. Intelligent landslide detection is a key research area in building LIMs, aiming to automatically or semi-automatically generate LIMs to reduce manual labor costs. In recent years, advancements in deep learning have led to successful applications of computer vision, which have provided automated solutions for the field of building LIMs^33,34^. In such automated landslide detection, the most critical components of AI-based and data-driven approaches are models and data, with the latter commonly referred to as landslide detection dataset (LDD). An LDD comprises a collection of trainable samples. Due to the limitations of AI models in processing excessively large images or other maps, these samples are typically derived by applying patch slicing to an image with broad extent, resulting in smaller patches with suitable size for models. In the context of semantic segmentation-based landslide detection, each sample includes both features and label. The features consist of optical imagery along with auxiliary layers such as multispectral bands, NDVI and DEM, while the label provides the boundary information of landslides aligned with these features. Automatically creating LIMs from scratch with AI models in unfamiliar regions requires the support of transfer learning, aiming to transfer knowledge from the source domain to the target domain^35^. LDDs provide source domain knowledge for AI models in this process, enabling automatic landslide detection tasks in similar or different regions^36^. Datasets like CAS, GDCLD, HRGLDD and Landslide4Sence provide good examples of LDDs^28,37–39^, but the overall scale of LDDs remains small, and rainfall-induced LDDs are relatively scarce.

**Fig. 1:**
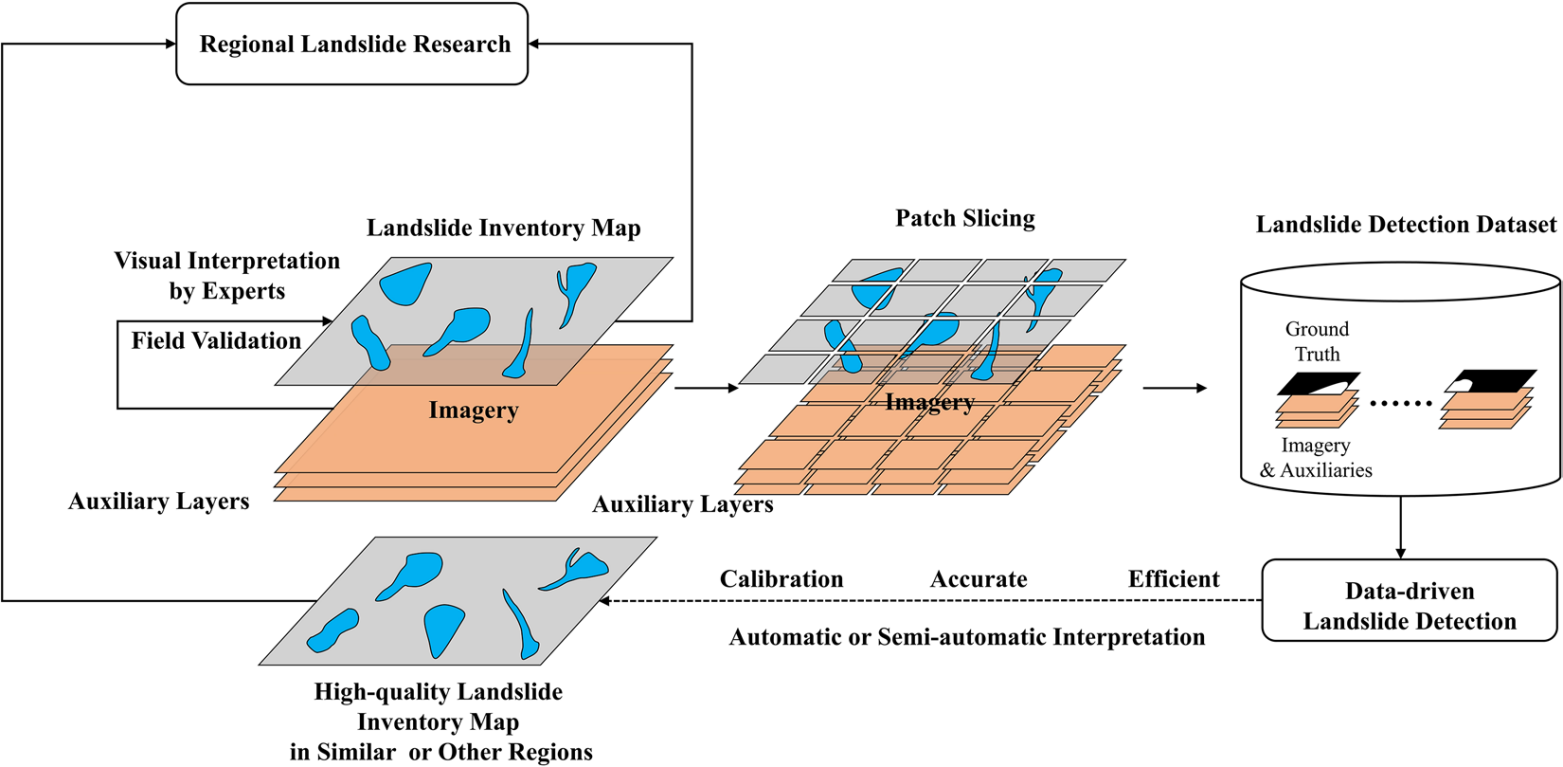
The generation process of LIM and LDD, as well as their links.

At the current stage, generating high-quality LIM primarily relies on expert visual interpretation and additional field validation, as automated or semi-automated methods in this domain remain underdeveloped and less reliable. Although numerous studies have examined automated and semi-automated landslide detection methods^40,41^, particularly those that are data-driven, most focus on model optimization and performance rather than on creating LIMs from scratch in unfamiliar regions. Currently, there are still bottlenecks in building LIMs automatically in unfamiliar regions using transfer learning techniques^42,43^, especially due to the scarcity of LDDs in source domains. Achieving generalization performance by data-driven approaches comparable to other data-sufficient fields such as autonomous driving or natural image analysis remains challenging. This further highlights the significance of constructing LDDs to fully achieve the automatic generation of high-quality LIMs. The links between LIM and LDD are illustrated in Fig. 1. In fact, LDDs are derivative products of high-quality LIMs, formed by combining and slicing LIMs with image and other layers. Since the ground truth (annotation or label, referring to the boundary of the target) in LDDs need to be as accurate as possible, the construction of an LDD requires a LIM that is highly aligned and precise with respect to the imagery and other layers before slicing into patches. Generating such a reliable LIM undoubtedly requires expert manual annotation and further field verification. Therefore, the generation of LDDs generally relies on high-quality LIMs, and the generated LDDs, in turn, promote the future automation of LIM generation based on transfer learning in similar or other regions.

**Fig. 2:**
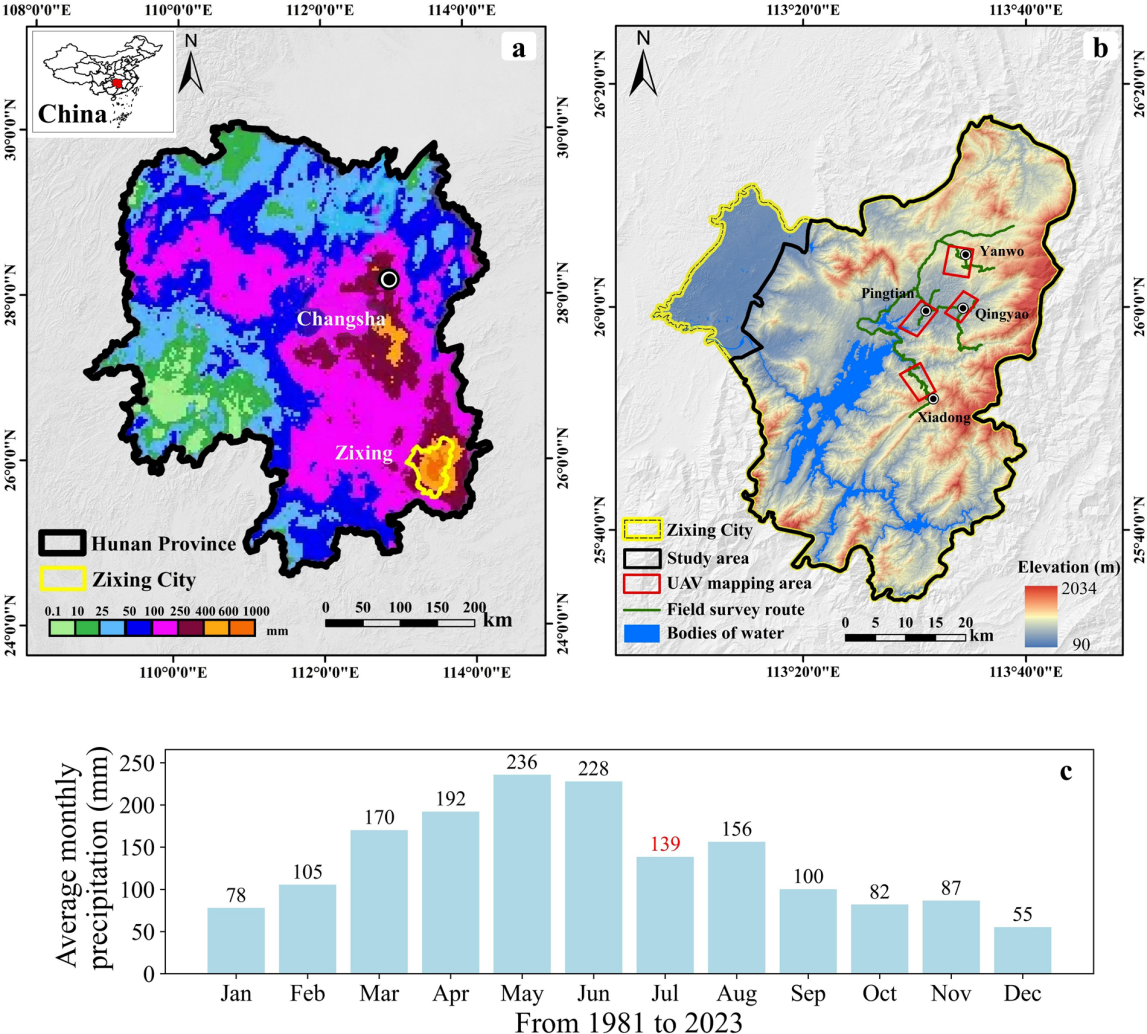
(**a**) The 5-day cumulative rainfall in Hunan Province, from July 24 to July 29, 2024 (source: China National Meteorological Information Center; https://weibo.com/1498396803/OpO4U7en2). It approximately represents the rainfall distribution in Hunan province during the Typhoon Gaemi. (**b**) Overview of the geographic location of the study area, field work route and UAV mapping. (**c**) Average monthly precipitation from 1981 to 2023 (source: National Centers for Environmental Information; https://www.ncei.noaa.gov).

**Fig. 3:**
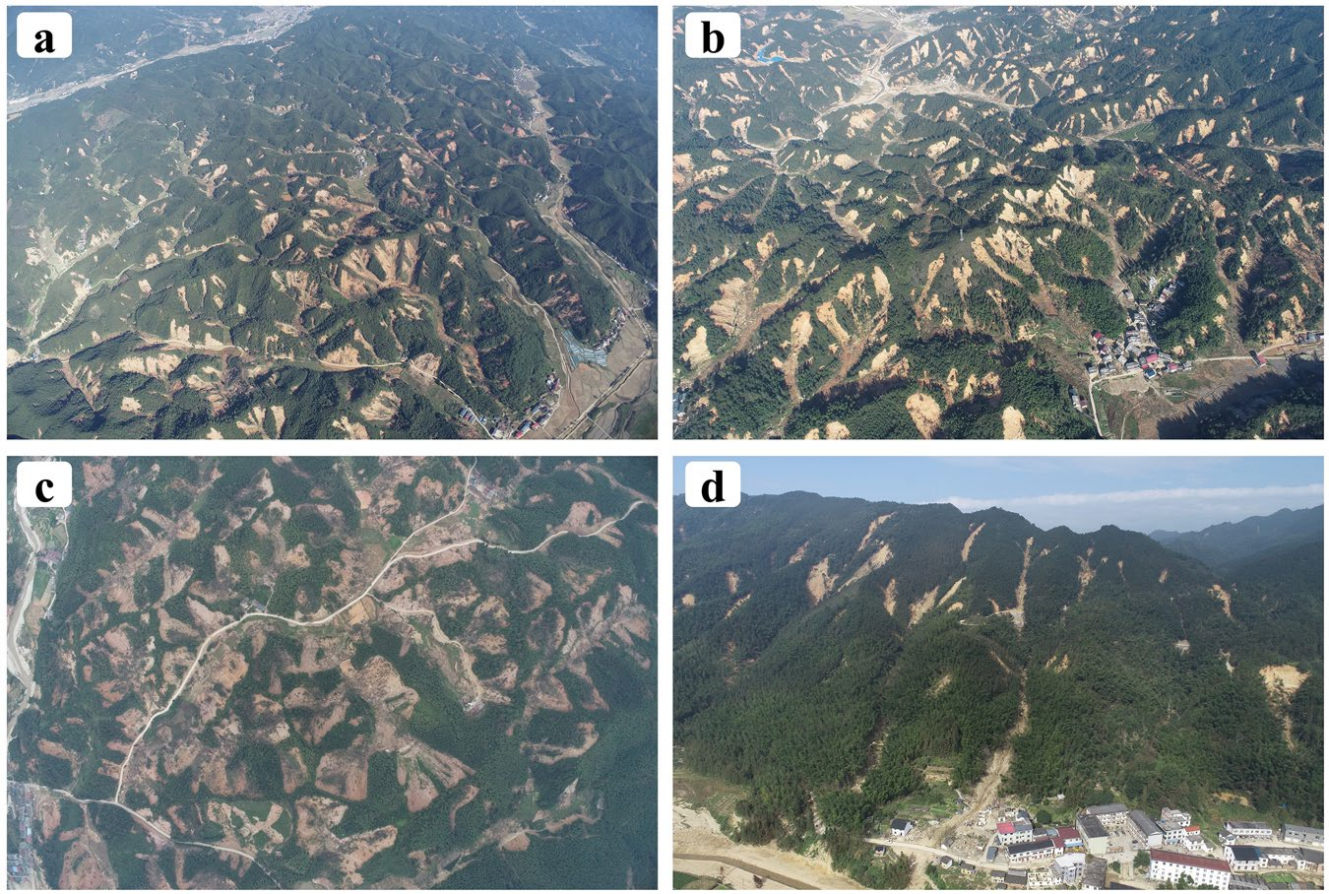
UAV field photos of clustered landslides in four villages severely affected by landslides in Zixing City, taken by DJI M300 equipped with lens of DJI L1. The approximate locations of these villages captured in the images could be seen in Fig. 1b. (**a**) Typical clustered landslides near Pingtian Village. (**b**) Typical clustered landslides near Yanwo Village. (**c**) Typical clustered landslides near Qingyao Village. (**d**) Typical clustered landslides near Xiadong Village, Bailang Town.

From July 26 to 28, 2024, Zixing, Hunan Province, China, experienced an extremely rare rainfall as a result of Typhoon Gaemi. Typhoon Gaemi made landfall along the Chinese coast and moved northwest while maintaining significant momentum^44^, resulting in heavy rainfall as it passed through Hunan Province. During this event, Zixing City recorded an average precipitation of 412.7 millimeters, with a maximum rainfall of 673.9 millimeters, far exceeding rainfall amounts in other regions of the province during the same period^45^ (Fig. 2a). Furthermore, based on reports from local residents, the rainfall associated with this event was considered exceptionally rare. We also collected monthly average rainfall data from 1981 to 2023 (Fig. 2c), which confirms that the event’s rainfall far exceeded historical averages. Within the area of Zixing City (Fig. 2b), the combined effects of floods and landslides triggered by this event affected nearly 128,000 people, destroyed over 11,800 houses, damaged nearly 600 roads, caused power outages for approximately 67,000 households, and impacted 210,000 acres of farmland^46,47^. Based on our findings, the region experienced at least 19,403 landslides, with certain areas being extremely densely developed (Fig. 3), which caused significant damage and hindered post-disaster rescue and reconstruction efforts. This paper describes the process of building the Rainfall-induced Landslide in Zixing (RLZX) datasets and the methods involved in data recording. The RLZX datasets primarily include a landslide inventory map (RLZX-LIM) and a landslide detection dataset (RLZX-LDD), which can be utilized for data-driven regional landslide research and intelligent landslide detection research, respectively. These datasets are created based on the interpretation of remotely sensed images, field investigations and UAV mapping to document the landslides triggered by the heavy rainfall in Zixing.

## Methods

**Fig. 4:**
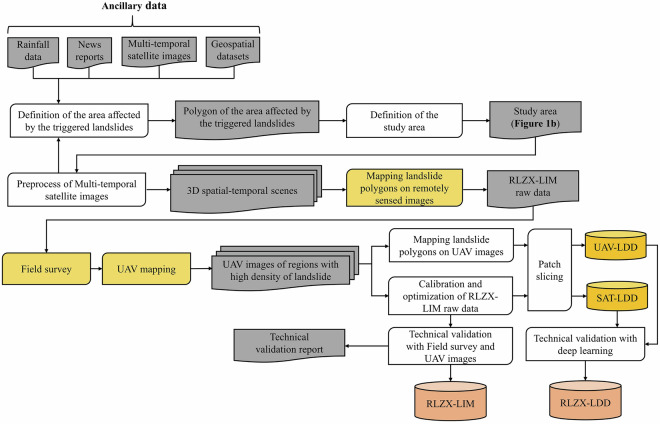
Flowchart of the method for building the RLZX datasets.

The RLZX datasets have been built following the process illustrated in Fig. 4. This procedure can be summarized in the following key stages: (i) preliminary data collection, data processing and definition of the study area; (ii) mapping landslide polygons based on 3D spatial-temporal scenes; (iii) field survey validation and UAV mapping; and (iv) generating RLZX-LDD and validation of the final versions of RLZX-LIM and RLZX-LDD.

### Available data

The data used in the process of mapping landslide polygons on remotely sensed images primarily consist of the optical satellite images before and after the rainfall event. We utilized satellite images produced by the China-made Gaofen satellite series as the interpretation base layers after the rainfall event. The chosen satellites produce panchromatic images with a resolution of 2 meters and multispectral images with a resolution of 8 meters. The multispectral bands include blue (B, 0.45–0.52 μm), green (G, 0.52–0.59 μm), red (R, 0.63–0.69 μm), and near-infrared (NIR, 0.77–0.89 μm). After performing radiometric calibration, FLAASH atmospheric correction, and RPC orthorectification on both images, we fused them using NNDiffuse pan sharpening to obtain the final multispectral image with a resolution of 2 m. The above operations are all mature functions in ENVI and the standardized processing methods for Gaofen imagery. A 12.5 m DEM from ALOS PALSAR was used in the orthorectification process. After performing 3D image and terrain overlay checks, the accuracy was found to be sufficiently high to meet the requirements of our task. For comparison, we used images from Sentinel-2 L2A with a resolution of 10 m to depict the earth's surface before the rainfall event. To address the high cloud content in the local atmosphere after the rainfall, we collected and mosaicked multi-temporal Gaofen images as close as possible in time. The post-rain images include Gaofen-1 images from August 3, 2024, and September 4, 2024, as well as Gaofen-6 images from August 5, 2024. The pre-rainfall images consist of Sentinel-2 L2A images acquired on March 10, 2023; July 22, 2023; November 20, 2023; and July 22, 2024. The multi-temporal pre-rainfall images aim to provide a detailed representation of the earth's surface just prior to the rainfall. All of the above images cover the entire extent of our study area.

To define the study area, ancillary data including relevant news reports, rainfall data (Fig. 2a) and various geospatial data have been collected. The geospatial data mainly consist of the administrative boundary of Zixing City (source: https://datav.aliyun.com/portal/school/atlas/area_selector), a 12.5 m DEM (source: https://search.asf.alaska.edu) and OpenStreetMap data (source: https://www.openstreetmap.org). Preliminary data compilation indicates that Zixing City is the region in Hunan Province experiencing the heaviest rainfall (Fig. 2a) and the most severe damage from landslides (according to the news reports), particularly in the eastern mountainous areas. A preliminary visual interpretation of satellite images covering Zixing City and its surroundings revealed that landslides are primarily concentrated within the administrative boundary of Zixing City, with very few observed outside of this boundary. Based on the aforementioned preliminary investigation, the study area is defined by the administrative boundaries of Zixing City, excluding the urban areas in the northwestern part of the city, where the terrain is relatively flat and received lower rainfall, as illustrated in Fig. 2b.

### Landslide inventory mapping

**Fig. 5:**
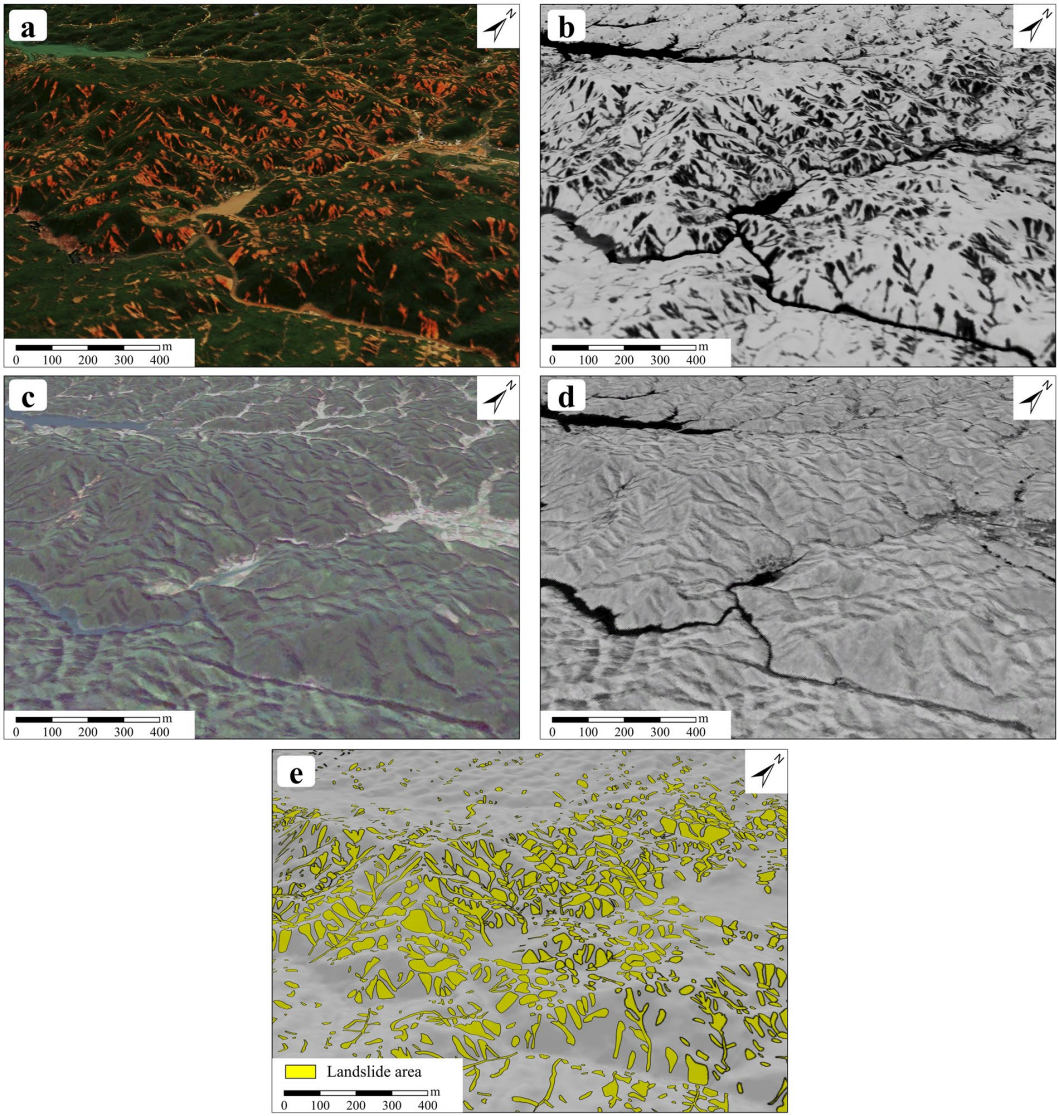
Excerpts of landslide inventory mapping process based on 3D spatial-temporal scenes. (**a,b**) Gaofen satellite images and corresponding NDVI after rainfall event. (**c,d**) Sentinel-2 satellite images and corresponding NDVI before rainfall event. (**e**) Interpretation results of Landslide areas based on comparison of multi-temporal optical images and NDVI with assistance of 3D terrain.

The process of building LIM was conducted using ArcGIS Pro 3.0 software. The mapping (annotation or labeling) of landslide polygons by visual interpretation primarily utilized its integrated 2D and 3D functionality, which allows for the simultaneous exploration of 2D and 3D scenes of the same area while creating and editing various files. The pre-rainfall images (Sentinel L2A images), post-rainfall images (Gaofen images) and UAV images could be overlaid on the 3D surface generated from the 12.5 m DEM from ALOS PLSAR to create 3D spatial-temporal scenes in ArcGIS Pro (Fig. 5a,c). These spatial-temporal scenes allow us to explore the earth's surface both spatially and across several time points before and after this event, from 3D perspectives. Additionally, we calculated the corresponding NDVI layers using the optical bands of NIR and R from the pre-rainfall and post-rainfall satellite images and overlaid them onto the 3D surface (Fig. 5d). NDVI can clearly reflect the surface vegetation coverage, which helps labelers better observe surface changes and aids in landslide interpretation. Finally, we focused on the changes in the pre-rainfall and post-rainfall RGB surface images to create landslide polygons on Gaofen images (post-rainfall), supplemented by identifying landslide terrain through DEM analysis and monitoring vegetation coverage via NDVI (Fig. 5e). This approach forms the basis for our LIM production method.

The generation of the LIM was accomplished through the collaborative efforts of multiple landslide interpretation experts with annotation experience and unified training. To ensure quality control, each expert’s labeling results underwent several rounds of review, during which issues such as misclassification, missed detections and boundary misalignment were corrected. Initial annotation work was conducted on satellite images. Following field surveys and UAV mapping (with the mapping area shown in Fig. 2b), detailed labeling of UAV images was performed, which was then followed by further optimization of the labeling results on the satellite images.

### Building the landslide detection datasets

As shown in Fig. 1, LDD is a collection of trainable pairs of ground truth of landslide, imagery and auxiliaries. Deep learning-based landslide detection models are unable to directly process broad extent remotely sensed or UAV images. As such, these datasets must be sliced into smaller patches, a process referred to as patch slicing. The patch slicing function of ArcGIS Pro was used to generate RLZX-LDD. This function automatically creates sliding windows around the landslide polygons. By applying numerous sliding windows to slice the base maps (landslide polygons and images), it produces classified patches consisting of pairs of images and their corresponding labels. This function eliminates the need for manual selection of the slicing area and can be directly applied to broad extent remotely sensed images. It automatically avoids generating a massive number of redundant non-landslide patches. The main hyperparameters of this process include the slicing size and slicing stride. A slicing size that is too large can result in the landslide target occupying a small proportion of the patch, making it difficult for the models to be trained. Conversely, a slicing size that is too small can cause the landslide target to become too large, hindering effective model deployment. The slicing stride controls the degree of overlap between generated patches. Overlapping patches contain a significant amount of the same content, which can lead to an substantial increase in the dataset volume. Based on our experience with other datasets, we set both the slicing size and slicing stride to 256 for satellite images and 1024 for UAV images. Additionally, a threshold of 0.3% for landslide area proportion was established to remove samples with very small targets. Through further data cleaning and format conversion, we obtained trainable LDD for deep learning. We applied the above workflow separately to generate the LDD for satellite images (SAT-LDD) and the LDD for UAV images (UAV-LDD). Together, SAT-LDD and UAV-LDD form the multi-source RLZX-LDD for the extreme rainfall event in Zixing.

### Deep learning models

To assess the quality and performance of our LDD for landslide detection using semantic segmentation techniques, we selected several commonly used and well-performing models. These include the convolution-based networks U^2^-Net^48^ and Deeplabv3+^49^, as well as transformer-based network Segformer^50^ and a lightweight transformer-convolution hybrid network MobileUNETR^51^.

## Data Records

The RLZX-related datasets have been uploaded to Figshare^52^ and are openly available for researchers in the field of regional landslides studies and intelligent landslide detection. These files provide a detailed LIM and a LDD of the 19,403 landslides triggered by extreme rainfall in Zixing City, China, in July 2024. They serve as important data sources for both data-driven regional landslide research and landslide detection studies. Descriptions of these files are provided in Table 1. The two main datasets are RLZX-LIM and RLZX-LDD.

**Table 1:** Description of the files contained in RLZX open access repository.

File/folder name	Format	Description
RLZX-LIM	Shapefile	Polygon record containing 19,403 landslides caused in Zixing, China, from July 26 to 28, 2024. The dataset is generated from the 3D visual interpretation of multi-temporal satellite images.
RLZX-LDD	Tiff and PNG	This dataset is composed of SAT-LDD (generated from satellite images) and UAV-LDD (generated from four UAV images). Both of them have the same file structure, which contains three subfolders: images, labels and DEM (consists of non-overlapping patches with matching names).
UAV_mapping	Tiff and Shapefile	Four UAV mosaic images of high-density landslide areas along with their corresponding landslide labels.
Field_logs	PDF	A landslide validation report based on field survey. This file records the occurrence of landslides in RLZX-LIM, along with corresponding field photos and satellite images for comparison.
Terrain_factors	Tiff	9 terrain parameter files that may be related to the landslide occurrence in the area.
Study_area	shapefile	The study area for this dataset. This area primarily limits the scope of our visual interpretation based on remote sensing images.

**Fig. 6:**
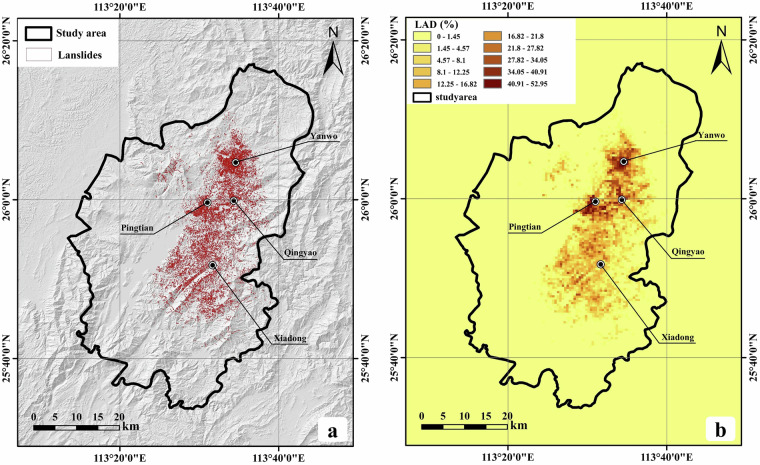
(**a**) The distribution of landslide polygons in RLZX-LIM. (**b**) The landslide area density in the RLZX-LIM.

**Fig. 7:**
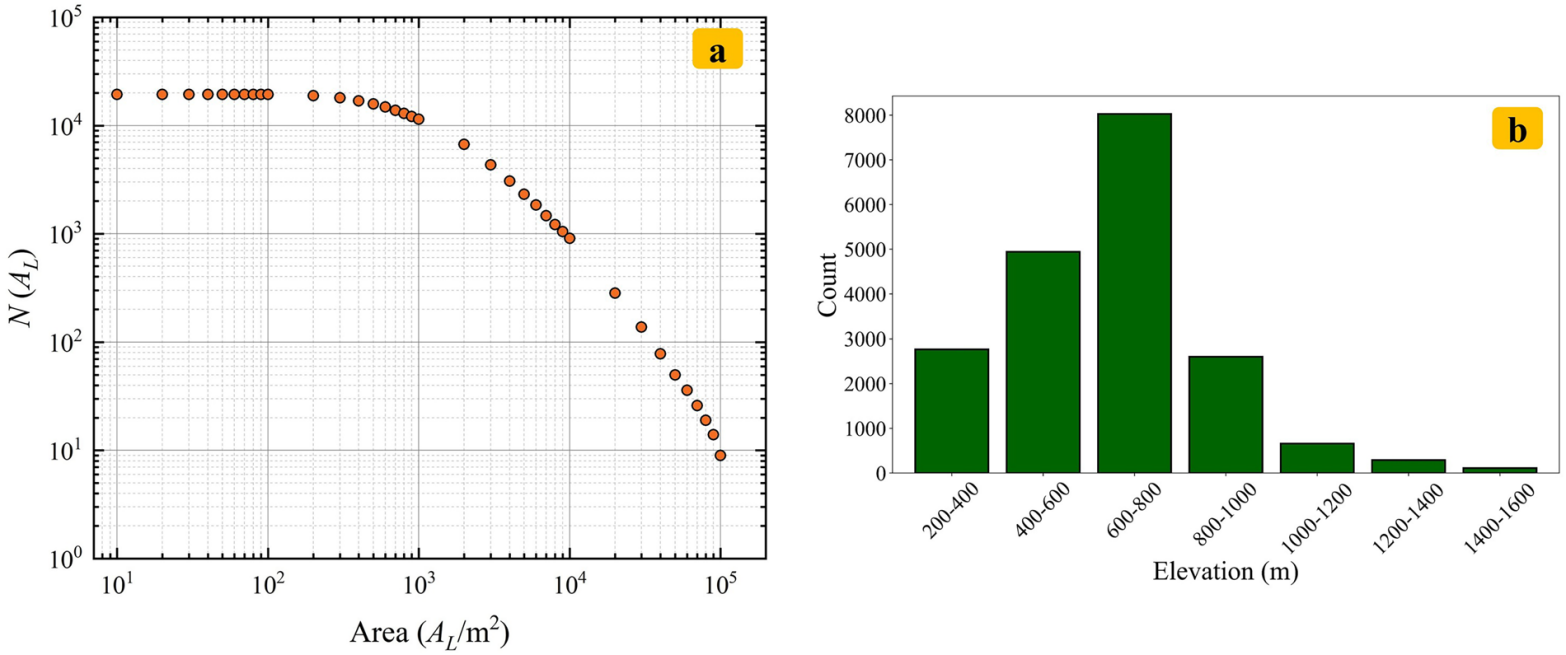
(**a**) Relationship of cumulative number of landslides and landslide area. The *N*(*A*_*L*_) refers to the number of landslides with an area greater than *A*_*L*_. (**b**) Relationship of landslide frequency and landslide elevation.

RLZX-LIM records 19,403 landslide polygons, which are stored in vector format as shapefile, as shown in Fig. 6a. We calculated the landslide area density (LAD) based on the proportion of landslide area within a 500 m × 500 m grid. Fig. 6b illustrates the LAD in the RLZX-LIM within the study area. Landslides are most densely distributed in the northeastern mountainous region of Zixing City, particularly around Pingtian Village, Qingyao Village and Yanwo Village, where the maximum LAD reaches 52.95%. The total area of landslide planar distribution in the region is 53.89 km^2^, with individual landslide planar areas ranging from 4 m^2^ to 171,982 m^2^. Fig. 7a illustrates the relationship between the cumulative number of landslides and landslide area. Fig. 7b depicts the relationship between landslide frequency and landslide elevation. There are strong power-law correlations between the cumulative number of landslides and the landslide area. A higher frequency of landslides is observed within the elevation range of 200 to 1,000 meters.

**Fig. 8:**
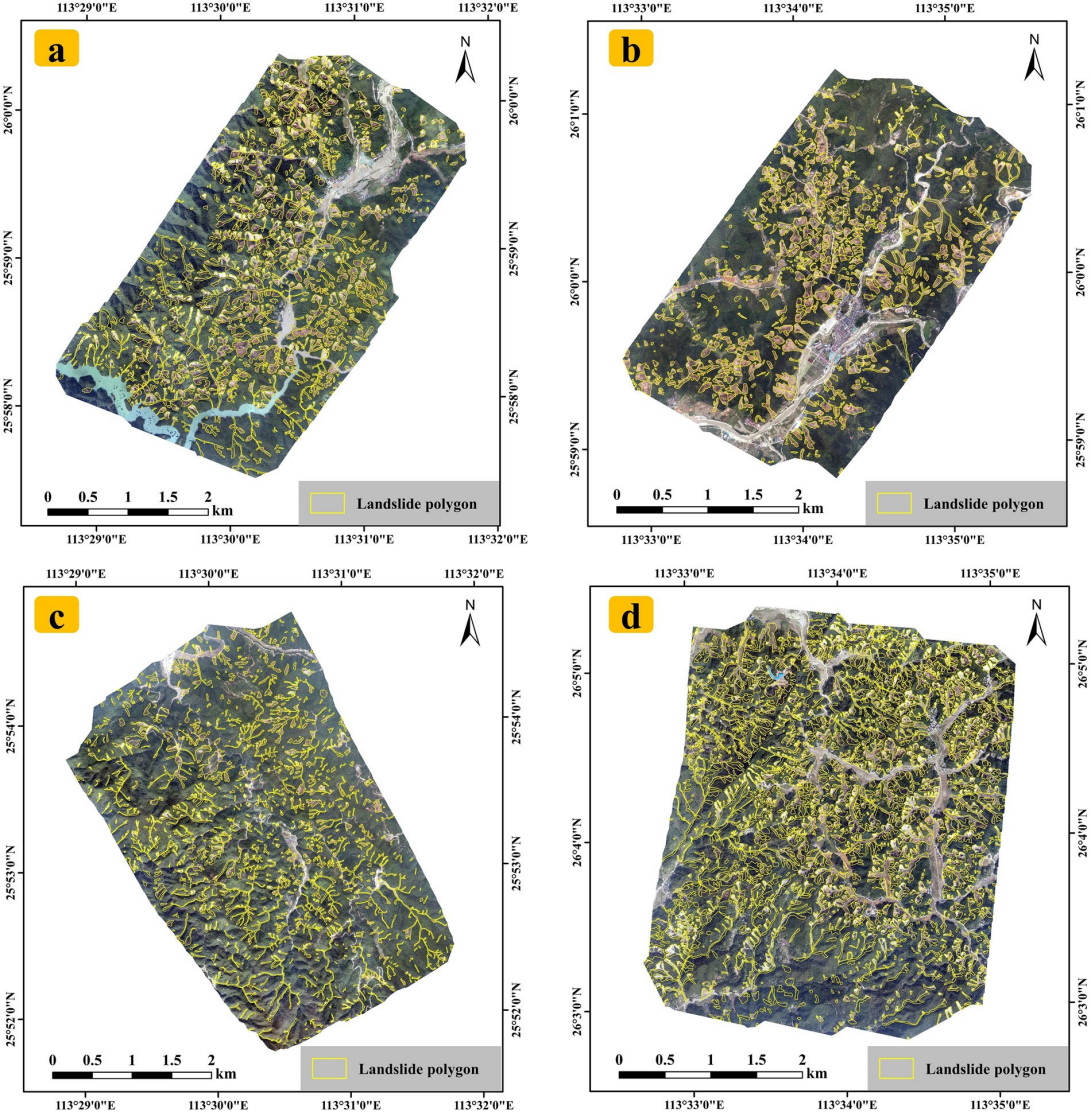
UAV images of the high-density landslide areas along with their corresponding landslide labels. (**a**) UAV image and corresponding landslide labels around Pingtian Village. (**b**) UAV image and corresponding landslide labels around Qingyao Village. (**c**) UAV image and corresponding landslide labels near Xiadong Village, Bailang Town. (**d**) UAV image and corresponding landslide labels around Yanwo Village.

RLZX-LDD is composed of two sub-datasets: SAT-LDD and UAV-LDD. The format and information of these two datasets are provided in Tables 2, 3. SAT-LDD contains image samples and corresponding labels generated from RLZX-LIM and Gaofen satellite images across the entire study area. UAV-LDD consists of image samples and corresponding labels derived from UAV images of four high-density landslide zones shown in Fig. 8. We have also matched these samples with slices of the DEM with a resolution of 12.5 m from ALOS PALSAR. Besides the patches that can be directly used for deep learning training, the UAV images and corresponding labels are also available for customized use, as shown in Fig. 8. The four UAV images cover areas of 14.91, 11.45, 15.59, and 15.66 km^2^, respectively. Their acquisition locations are shown in Fig. 2b. They feature a 0.3 m resolution and contain red, green and blue channels. Due to data restrictions, we are unable to provide the original satellite imagery directly. However, users can conveniently obtain landslide detection training samples derived from Gaofen imagery by using our SAT-LDD. In addition, we also encourage users to combine our released LIM and UAV images with satellite imagery from other sources to generate alternative versions of LDD for this event.

**Table 2:** Detailed information of RLZX-LDD.

Dataset	Number of samples	Patch size	Image channels	Resolution (m)	Proportion of landslide pixels (%)
SAT-LDD	2848	256*256	(B,G,R,NIR)	2	7.16
UAV-LDD	648	1024*1024	(R,G,B)	0.3	18.33

The RLZX-LDD is composed of the satellite version of SAT-LDD and the UAV version of UAV-LDD.

**Table 3:** The sample composition of RLZX-LDD.

Dataset	Image	Label	DEM
SAT-LDD			
UAV-LDD

To further expand the RLZX-related data, terrain factors potentially associated with rainfall-induced landslides are calculated using the ALOS 12.5 m DEM. These factors are available in the RLZX as raster layers.

## Technical Validation

### Validation of the RLZX-LIM

The validation of RLZX-LIM requires establishing a baseline. Obtaining the true count, geographic location, shape, and size of landslides on-site is extremely challenging. In our work, the road-aligned field survey and UAV mapping in the on-site investigation cover specific areas. On the one hand, data obtained (landslide cases in field logs and high-resolution UAV images) are highly reliable combined with expert experience. On the other hand, RLZX-LIM is derived from the visual interpretation of 2 m satellite images, which introduces certain errors due to low resolution, terrain correction inaccuracies and other factors during the cataloging process. In contrast, the landslide information established based on field surveys has higher reliability. Therefore, we use road-aligned field logs and landslide polygons annotated based on UAV images as the reference baseline for evaluating RLZX-LIM. These field data have limited coverage and serve only as a reference for evaluating RLZX-LIM or even SAT-LDD.

After preparing the final version of RLZX-LIM, its validation was conducted, covering the following aspects: (i) completeness and correctness; and (ii) geographic accuracy. The validation tasks were entirely based on our field survey, during when the data was collected in the forms of UAV mapping, road-aligned field investigation. Completeness is defined as the ratio between the number of landslides recorded in the RLZX-LIM and the total number of landslides observed through ground-based investigations (including road-aligned field investigation and UAV mapping). This metric indicates the overall integrity of the RLZX inventory and its tendency to omit landslides. Correctness indicates the proportion of landslides in the RLZX-LIM that were correctly annotated according to ground-based investigations, reflecting the accuracy of the RLZX-LIM and its error rate. Geographic accuracy refers to the deviations in the RLZX-LIM regarding the geographic location, geometric shape, and size of landslides.

**Fig. 9:**
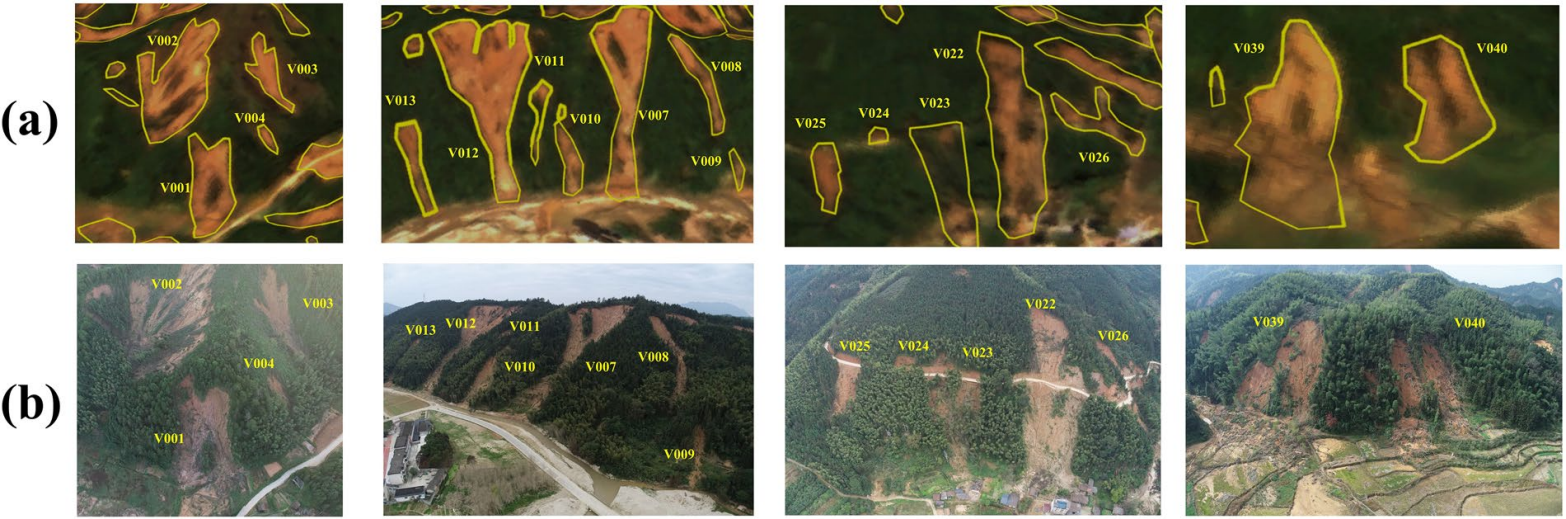
Examples of the comparison between RLZX-LIM and actual landslide in filed logs. The yellow mark is the on-site landslide number recorded in the field logs. (**a**) 3D scenes of RLZX-LIM on remotely sensed images. (**b**) field photos in field logs.

The completeness and correctness of RLZX-LIM have been validated through field investigations, with the survey route shown in Fig. 2b. This route was designed mainly considering road accessibility. Although random sampling would be a more preferable method for validating RLZX-LIM, most of the selected areas are inaccessible, making this approach impractical. Most of the validated landslides are along roads, which introduces a certain degree of bias into the validation results. Therefore, we declare that the validation results presented here primarily reflect the quality of the landslide records along roads in RLZX-LIM. For the overall quality, these results from the road-aligned survey should be considered as a reference rather than a holistic assessment. Along the survey route, approximately 1.1% of the landslides from RLZX-LIM were documented through photographs and spot markings for validation. A total of 213 landslides were recorded during the road-aligned investigation. These landslides were randomly selected along the roads, and we documented them using UAV and cameras. We verified completeness by comparing these landslides with those included in RLZX-LIM, and confirmed their correctness during the road-aligned survey. Detailed information is available in the “Field logs” of the data repository. According to the “Field logs,” the completeness of RLZX-LIM for the sampled landslides along the survey route is 96.71%. During the road-aligned survey, there were no false positives observed in RLZX-LIM, resulting in a correctness of 100%. The field photos correspond well with the remotely sensed images as shown in Fig. 9, demonstrating a reasonable level of correspondence between the landslide geometry in RLZX-LIM and the actual geometry. A certain extent in terms of geographic accuracy can be observed from the alignment between the landslide geometry in RLZX-LIM and actual geometry near the road.

The four UAV images are used to validate RLZX-LIM. The mapping areas of these images are based on our prior assessment of the RLZX-LIM raw data, covering the four sub-regions with the highest landslide density. The resolution of the UAV images (0.3 m) is significantly higher than that of the Gaofen satellite images (2 m) used in the creation of RLZX-LIM, allowing experts to easily and accurately identify landslides. The landslide polygons obtained from the annotated UAV images (using the same method as in the creation of RLZX-LIM) served as a reference for validating RLZX-LIM. The completeness and correctness of RLZX-LIM in these four regions are shown in Table 4. Geographic discrepancies exist at the data source level due to differences in production methods between UAV and satellite images, leading to similar offsets in the labels of both types of images. After excluding this inherent offset by manual calibration, we observed that the overlapping area of landslide polygons from different sources is generally near 80%. Measuring geographic accuracy is a complex issue, we have quantified and simplified it here. To further quantify the geographic accuracy of RLZX-LIM, we used the landslide polygons from four UAV images as the reference and identified the corresponding landslide polygons in RLZX-LIM. We randomly selected 50 pairs of landslide instances from each UAV image (one from the annotation on the UAV image and the other from the annotation on the Gaofen image), calculated the area differences between them, and divided these differences by the area of the landslide polygon from the UAV image as the bias rate. The average of these bias rates was used as a reference for the geometric bias rate of RLZX within the area covered by each UAV image. This geometric bias rate approximately represents the deviation between the landslide sizes in RLZX-LIM and the actual field conditions. The geometric bias rates of RLZX-LIM within the areas covered by the four UAV images are 21.7%, 20%, 27.7% and 21.2%, respectively. Since the UAV images were captured three months after the satellite images, discrepancies in the landslide polygons from different sources can be observed due to external factors such as debris removal and changes to the earth’s surface. These discrepancies introduce a certain amount of bias into our validation process of RLZX-LIM.

**Table 4:** The validation results of field logs and UAV images.

Validation data	Missing count	Error count	Total record count	Completeness (%)	Correctness (%)
Field logs	7	0	213	96.7	100
UAV_1	47	30	963	95.1	96.7
UAV_2	44	8	702	93.7	98.8
UAV_3	162	23	1059	84.7	97.4
UAV_4	79	26	1536	94.9	98.2

Based on field investigations and validation using UAV images, the quality of RLZX-LIM has been thoroughly confirmed through quantitative evaluation. According to Table 4, the completeness and correctness of RLZX-LIM in field logs and UAV images achieve relatively high values, which provide reference for the cases of missing records and wrong records in RLZX-LIM. Random sampling of RLZX-LIM in the areas of four UAV images shows that the area deviation rate is around 20%. Due to the low resolution comparing to UAV’s and uncertainties in terrain correction of satellite images, the geometric shape and area of the landslide polygons marked on them may deviate from the actual situation. Although the dataset records 19,403 landslides, we still consider it incomplete due to the limitations of satellite image resolution and the invisibility of many landslides caused by vegetation cover and shadows in remotely sensed images. We have made RLZX-LIM publicly available. It is worth emphasizing that this dataset is entirely based on remotely sensed images and manual labeling.

### Validation of the RLZX-LDD

RLZX-LDD is a dataset designed for intelligent landslide detection using deep learning and semantic segmentation techniques. Its quality will be assessed through deep learning models, with performance metrics reflecting this evaluation. This is a fundamental aspect of machine learning, as models learn from the data provided to them. When the dataset contains low-quality noise samples, the model’s performance suffers. Conversely, higher quality data enables the model to identify meaningful patterns, resulting in improved performance. This validation approach is also employed by other landslide detection datasets, such as CAS^37^, HRGLDD^39^, GDCLD^38^, and Landslide4Sense^28^.

The U^2^-Net^48^, Deeplabv3+^49^, Segformer^50^, and MobileUNETR^51^ were utilized to validate the quality of RLZX-LDD for the task of landslide segmentation. In the deep learning training process, we used PyTorch as the framework, set the batch size to 16, and employed the AdamW optimizer (beta1 = 0.9, beta2 = 0.999). A hybrid loss function combining focal loss and dice loss was implemented to address the detection of imbalanced landslide class. We adopted a warm-up and cosine annealing strategy, with the maximum learning rate set to 5e-4 and the minimum learning rate set to 1e-6. During epochs 0 to 10, the learning rate increases linearly from the minimum to the maximum. Between epochs 10 and 200, the learning rate gradually decays from the maximum to the minimum using cosine annealing. After epoch 200, the learning rate remains at the minimum, and an early stopping strategy is applied to terminate the training during this period. The maximum number of epochs is set to 300. All images are resized to 256 as input to the model, and the data augmentation strategies of horizontal inversion, vertical inversion, random cropping, rotation, Gaussian noise, Gaussian blur and random brightness contrasting are used. All training was completed on the RTX 4090 GPU.

To validate the quality of our dataset, we split the training and testing set with a ratio of 7:3, the most commonly used ratio in intelligent landslide detection. Our samples are sliced in a non-overlapping manner, ensuring that there is no information leakage during this process. The four bands of R, G, B, and DEM are used as inputs, as research has confirmed the boosting effect of DEM on landslide detection^36,53,54^. Precision, recall, mIoU and F1 score are selected as the evaluation metrics for model performance, all of these metrics are commonly used in the tasks of landslide segmentation^55^. The validation results of RLZX-LDD are shown in Table 5. The mIoU range for all models fluctuated between 73.16% and 79.91%, indicating the high quality of the whole dataset. Among four models, U^2^-Net consistently achieved the best performance with the highest mIoU of 79.91% in UAV-LDD, followed by MobileUNETR, Segformer and Deeplabv3 + . The models trained on UAV-LDD generally achieved the highest accuracy, followed by models trained by the hybrid dataset, while the models trained on SAT-LDD have the lowest performance. This indicates that the quality of UAV-LDD is higher than that of the SAT-LDD, which may result from the high-resolution images and high-quality labeling. It is worth noting that the hybrid UAV-LDD and SAT-LDD dataset can help the models achieve relatively excellent performance. This phenomenon is also observed in the CAS and GDCLD dataset, highlighting the superiority of combining UAV and satellite image samples.

**Table 5:** The validation results of the quality of RLZX-LDD.

Dataset	Model	Precision (%)	Recall (%)	F1 score (%)	mIoU (%)
UAV-LDD	Deeplabv3+^49^	76.72	75.67	76.19	75.90
	Segformer^50^	73.22	72.72	72.97	73.21
	MobileUNETR^51^	78.77	75.13	76.90	76.60
	U^2^-Net^48^	80.79	80.84	80.82	79.91
SAT-LDD	Deeplabv3+^49^	73.75	64.07	68.57	73.82
	Segformer^50^	70.59	64.95	67.65	73.16
	MobileUNETR^51^	75.40	63.12	68.72	73.95
	U^2^-Net^48^	77.89	63.02	69.67	74.61
UAV-LDD & SAT-LDD (RLZX-LDD)	Deeplabv3+^49^	77.03	68.33	72.42	75.79
	Segformer^50^	78.40	68.30	73.01	76.23
	MobileUNETR^51^	78.60	68.36	73.12	76.32
	U^2^-Net^48^	79.80	71.66	75.51	78.01

### Comparative experiments of RLZX-LDD and other datasets on transfer learning

The introduction of RLZX-LDD is largely driven by the need for cross-regional landslide detection. Cross-regional landslide detection refers to the task of performing intelligent landslide detection in a new and unfamiliar area using deep learning models, where local data is scarce and unavailable, making it hard to conduct the task directly due to the dependency of deep learning models on annotated data. In such cases, the model is trained using data from other regions, transferring the knowledge learned from those areas to apply to this unfamiliar region. This process is also known as transfer learning. In the field of transfer learning, data from other regions is referred to as the source domain dataset. This type of data is crucial for future cross-regional landslide detection tasks. The generalization ability brought to the model by RLZX-LDD as a source domain dataset needs further testing.

The model’s generalization ability and robustness brought by the dataset are further tested using all data from RLZX-LDD for the task of cross-regional landslide detection. In this experiment, the Longxi River sub-dataset from the CAS dataset is selected as the target domain for transfer learning, which contains 1,769 satellite image samples and 2,504 UAV image samples^37^. In addition, three other regions in the CAS dataset (Mengdong, Jiuzhai Valley and Moxitaidi) and the Bijie dataset are selected for comparison with our RLZX-LDD^36,37^. Longxi River, Mengdong, Jiuzhai Valley, Moxitaidi, and Bijie are all landslide detection datasets that are consistent with RLZX-LDD. Longxi River, Jiuzhai Valley, and Moxitaidi are located in different regions of Sichuan Province, China, while Mengdong is located in Yunnan Province and Bijie is in Guizhou Province. These datasets, like RLZX-LDD, can be used as source domain datasets for landslide detection, providing valuable information for transfer learning models. The quality of these datasets can be referenced by the model’s performance, which reflects the amount of useful information they contain. The common RGB channels of these datasets were selected as the input. We adopted a transfer learning strategy similar to Bhuyan^43^ and Dong^42^, applying the same pre-training settings on source domains and fine-tuning settings on target domain. All data from the datasets selected for comparison (source domains) are utilized in the pre-training stage, while only 10% of the data from the Longxi River (target domain) is used for fine-tuning. The performances of models trained on different source domain datasets are tested on the remaining 90% of the data in the target domain. The pre-training and fine-tuning settings are consistent with previous training. We have selected the best-performing U^2^-Net to evaluate the generalization ability of models trained by different datasets.

The comparative results are shown in Table 6. The RLZX-LDD dataset achieves the highest F1 score (74.4%) and mIoU (76.23%), demonstrating its superior quality compared to other datasets on the cross-regional tasks. The Moxitaidi dataset achieves the second-best performance, and is also composed of both UAV and satellite image samples. The other three datasets, due to their single source, data volume, data quality or feature differences, performed inferior to the two hybrid datasets. The triggers of landslides (earthquake or rainfall), the image source and the differences in regional landcover features can also affect the cross-regional performance of landslide detection. The target domain Longxi River dataset is also a rainfall-induced landslide dataset, which could be one of the reasons why RLZX-LDD performs the best. Most landslide detection datasets like CAS and GDCLD focus more on co-seismic landslides, while rainfall-induced landslide datasets are scarce as RLZX-LDD effectively helps narrow this gap. The above comparison highlights the excellent robustness and potential generalization ability brought by RLZX-LDD.

**Table 6:** The comparative results of the generalization ability of models training on different datasets.

Model	Dataset	Number of samples	Image source	Precision (%)	Recall (%)	F1 score (%)	mIoU (%)
U^2^-Net^48^	Bijie^37^	770	UAV	73.17	64.67	68.66	72.27
	Mengdong^36^	1155	SAT	73.84	69.21	71.45	74.15
	Jiuzhai Valley^36^	7677	UAV	72.34	70.85	71.59	74.17
	Moxitaidi^36^	2119	SAT & UAV	73.98	69.64	71.74	74.36
	RLZX-LDD	3496	SAT & UAV	74.41	74.4	74.4	76.23

## Usage Notes

The RLZX dataset aims to support researchers in two major areas of data-driven regional landslide research (susceptibility, hazard and risk assessment), as well as landslide detection. It consists of RLZX-LIM and RLZX-LDD, which contain 19,403 landslide polygons and 3,496 landslide image samples from UAV and satellites, respectively. The substantial size of these datasets makes them particularly suitable for machine learning-based regional research on landslides. It is important to note that RLZX-LIM may still be incomplete due to resolution limitations and the invisibility of many landslides, especially smaller ones. In this article, we have provided quantitative evaluation references for the quality of RLZX-LIM based on field data (road-aligned field logs and UAV mapping). RLZX-LDD is created from Gaofen images and high-resolution UAV images, enabling models to achieve competitive performance and enhanced generalization compared to other single-region LDDs mentioned in this paper (three sub-regions in the CAS dataset and the Bijie dataset). Additionally, RLZX-LDD retains the NIR band from the satellite images, which can be used to calculate metrics like NDVI. More importantly, the DEM band has also been included, which is not available in many other datasets. This dataset can effectively assist in landslide detection by integrating optical and topographic information. It is also recommended to combine our RLZX datasets with other datasets for cross-regional or large-scale research, in line with the emerging trend of big data in landslide studies.

## Data Availability

The code for loading dataset, data augmentation and other relevant information have been available on GitHub: https://github.com/klaus2023/RLZX-landslide-inventory-and-landslide-detection-datasets.

## References

[1] Guzzetti, F. *et al*. Landslide inventory maps: New tools for an old problem. *Earth-Science Reviews* **112**, 42–66 (2012).

[2] Koks, E. E. *et al*. A global multi-hazard risk analysis of road and railway infrastructure assets. *Nat Commun* **10**, 2677 (2019). 31239442 10.1038/s41467-019-10442-3 PMC6592920

[3] Keefer, D. K. Landslides caused by earthquakes. *GSA Bulletin* **95**, 406–421 (1984).

[4] Collins, B. D. & Znidarcic, D. Stability Analyses of Rainfall Induced Landslides. *Journal of Geotechnical and Geoenvironmental Engineering* **130**, 362–372 (2004).

[5] McColl, S. T. Landslide causes and triggers. in *Landslide hazards, risks, and disasters* 13–41 (Elsevier, 2022).

[6] Gariano, S. L. & Guzzetti, F. Landslides in a changing climate. *Earth-Science Reviews* **162**, 227–252 (2016).

[7] Wang, F. *et al*. The Hongchi landslide triggered by heavy rainfall from Super Typhoon In-Fa on 25 July 2021 in Hangzhou City, Zhejiang Province, China. *Bull Eng Geol Environ* **81**, 411 (2022).

[8] Wang, F. *et al*. The Wuxie debris flows triggered by a record-breaking rainstorm on 10 June 2021 in Zhuji City, Zhejiang Province, China. *Landslides* **19**, 1913–1934 (2022).

[9] Trigila, A., Iadanza, C. & Spizzichino, D. Quality assessment of the Italian Landslide Inventory using GIS processing. *Landslides* **7**, 455–470 (2010).

[10] Dou, J. *et al*. Different sampling strategies for predicting landslide susceptibilities are deemed less consequential with deep learning. *Science of The Total Environment* **720**, 137320 (2020). 32325551 10.1016/j.scitotenv.2020.137320

[11] Fang, Z. *et al*. Speech-recognition in landslide predictive modelling: A case for a next generation early warning system. *Environmental Modelling & Software* **170**, 105833 (2023).

[12] Fu, Z. *et al*. An Integrated Framework of Positive-Unlabeled and Imbalanced Learning for Landslide Susceptibility Mapping. *IEEE Journal of Selected Topics in Applied Earth Observations and Remote Sensing* **17**, 15596–15611 (2024).

[13] Tang, G., Fang, Z. & Wang, Y. Global landslide susceptibility prediction based on the automated machine learning (AutoML) framework. *Geocarto International* (2023).

[14] Fang, Z., Wang, Y., van Westen, C. & Lombardo, L. Space-time modeling of landslide size by combining static, dynamic, and unobserved spatiotemporal factors. *CATENA* **240**, 107989 (2024).

[15] Ullah, K. *et al*. Spatiotemporal dynamics of landslide susceptibility under future climate change and land use scenarios. *Environ. Res. Lett.* **19**, 124016 (2024).

[16] Wang, T. *et al*. From spatio-temporal landslide susceptibility to landslide risk forecast. *Geoscience Frontiers* **15**, 101765 (2024).

[17] Fang, Z., Wang, Y., van Westen, C. & Lombardo, L. Landslide hazard spatiotemporal prediction based on data-driven models: Estimating where, when and how large landslide may be. *International Journal of Applied Earth Observation and Geoinformation* **126**, 103631 (2024).

[18] Fu, Z., Wang, F., Dou, J., Nam, K. & Ma, H. Enhanced Absence Sampling Technique for Data-Driven Landslide Susceptibility Mapping: A Case Study in Songyang County, China. *Remote Sensing* **15**, 3345 (2023).

[19] Nguyen, B.-Q.-V. & Kim, Y.-T. Regional-scale landslide risk assessment on Mt. Umyeon using risk index estimation. *Landslides* **18**, 2547–2564 (2021).

[20] Guzzetti, F., Reichenbach, P., Cardinali, M., Galli, M. & Ardizzone, F. Probabilistic landslide hazard assessment at the basin scale. *Geomorphology* **72**, 272–299 (2005).

[21] Van Westen, C. J., Castellanos, E. & Kuriakose, S. L. Spatial data for landslide susceptibility, hazard, and vulnerability assessment: An overview. *Engineering geology* **102**, 112–131 (2008).

[22] Van Den Eeckhaut, M. & Hervás, J. State of the art of national landslide databases in Europe and their potential for assessing landslide susceptibility, hazard and risk. *Geomorphology* **139–140**, 545–558 (2012).

[23] Martha, T. R., van Westen, C. J., Kerle, N., Jetten, V. & Vinod Kumar, K. Landslide hazard and risk assessment using semi-automatically created landslide inventories. *Geomorphology* **184**, 139–150 (2013).

[24] Santangelo, M. *et al*. Inventory of landslides triggered by an extreme rainfall event in Marche-Umbria, Italy, on 15 September 2022. *Sci Data* **10**, 427 (2023). 37400466 10.1038/s41597-023-02336-3 PMC10318071

[25] Reichenbach, P., Rossi, M., Malamud, B. D., Mihir, M. & Guzzetti, F. A review of statistically-based landslide susceptibility models. *Earth-Science Reviews* **180**, 60–91 (2018).

[26] Ghorbanzadeh, O., Meena, S. R., Blaschke, T. & Aryal, J. UAV-Based Slope Failure Detection Using Deep-Learning Convolutional Neural Networks. *Remote Sensing* **11**, 2046 (2019).

[27] Zhang, R., Lv, J., Yang, Y., Wang, T. & Liu, G. Analysis of the impact of terrain factors and data fusion methods on uncertainty in intelligent landslide detection. *Landslides* (2024).

[28] Ghorbanzadeh, O., Xu, Y., Ghamisi, P., Kopp, M. & Kreil, D. Landslide4Sense: Reference Benchmark Data and Deep Learning Models for Landslide Detection. *IEEE Trans. Geosci. Remote Sensing* **60**, 1–17 (2022).

[29] Fu, L. *et al*. Detecting slow-moving landslides using InSAR phase-gradient stacking and deep-learning network. *Frontiers in Environmental Science* **10** (2022).

[30] van Natijne, A. L., Bogaard, T. A., van Leijen, F. J., Hanssen, R. F. & Lindenbergh, R. C. World-wide InSAR sensitivity index for landslide deformation tracking. *International Journal of Applied Earth Observation and Geoinformation* **111**, 102829 (2022).

[31] Su, Z. *et al*. Deep convolutional neural network–based pixel-wise landslide inventory mapping. *Landslides* **18**, 1421–1443 (2021).

[32] Xu, Q. *et al*. Remote sensing for landslide investigations: A progress report from China. *Engineering Geology* **321**, 107156 (2023).

[33] Mohan, A., Singh, A. K., Kumar, B. & Dwivedi, R. Review on remote sensing methods for landslide detection using machine and deep learning. *Transactions on Emerging Telecommunications Technologies* **32**, e3998 (2021).

[34] Catani, F. Landslide detection by deep learning of non-nadiral and crowdsourced optical images. *Landslides* **18**, 1025–1044 (2021).

[35] Wu, L. *et al*. Landslide mapping based on a hybrid CNN-transformer network and deep transfer learning using remote sensing images with topographic and spectral features. *International Journal of Applied Earth Observation and Geoinformation* **126**, 103612 (2024).

[36] Ji, S., Yu, D., Shen, C., Li, W. & Xu, Q. Landslide detection from an open satellite imagery and digital elevation model dataset using attention boosted convolutional neural networks. *Landslides* **17**, 1337–1352 (2020).

[37] Xu, Y. *et al*. CAS Landslide Dataset: A Large-Scale and Multisensor Dataset for Deep Learning-Based Landslide Detection. *Sci Data* **11**, 12 (2024). 38168493 10.1038/s41597-023-02847-z PMC10762236

[38] Fang, C. *et al*. A globally distributed dataset of coseismic landslide mapping via multi-source high-resolution remote sensing images. *Earth System Science Data Discussions* 1–42 (2024).

[39] Meena, S. R. *et al*. HR-GLDD: a globally distributed dataset using generalized deep learning (DL) for rapid landslide mapping on high-resolution (HR) satellite imagery. *Earth System Science Data* **15**, 3283–3298 (2023).

[40] Chen, T. *et al*. BisDeNet: A New Lightweight Deep Learning-based Framework for Efficient Landslide Detection. *IEEE Journal of Selected Topics in Applied Earth Observations and Remote Sensing* 1–17 (2024).

[41] Lv, P., Ma, L., Li, Q. & Du, F. ShapeFormer: A Shape-Enhanced Vision Transformer Model for Optical Remote Sensing Image Landslide Detection. *IEEE Journal of Selected Topics in Applied Earth Observations and Remote Sensing* **16**, 2681–2689 (2023).

[42] Dong, A. *et al*. Accelerating Cross-Scene Co-Seismic Landslide Detection Through Progressive Transfer Learning and Lightweight Deep Learning Strategies. *IEEE Transactions on Geoscience and Remote Sensing* **62**, 1–13 (2024).

[43] Bhuyan, K. *et al*. Generating multi-temporal landslide inventories through a general deep transfer learning strategy using HR EO data. *Sci Rep* **13**, 162 (2023). 36599911 10.1038/s41598-022-27352-y PMC9813262

[44] Fraser-Baxter, S. Climate change increased Typhoon Gaemi’s wind speeds and rainfall, with devastating impacts across the western Pacific region | Policy Commons.

[45] Chenzhou Municipal People’s Government Press Conference, Answers to Journalists’s Questions. *Chenzhou Daily*, 001 (2024).

[46] Zhang, H., Yang, J., Liang, K., & Luo, H. News conference held in Chenzhou to report on disaster and relief situation in Zixing. *Hunan Daily*, 003 (2024).

[47] Zixing City’s affected administrative villages have preliminarily achieved access to roads, electricity, communication, and water, and post-disaster reconstruction is in full swing. 50 confirmed dead and 15 missing. *WeChat Public Platform*, https://mp.weixin.qq.com/s/BnTujKuo-cFAGDu2EA4beg.

[48] Qin, X. *et al*. U2-Net: Going deeper with nested U-structure for salient object detection. *Pattern Recognition* **106**, 107404 (2020).

[49] Chen, L.-C., Zhu, Y., Papandreou, G., Schroff, F. & Adam, H. Encoder-Decoder with Atrous Separable Convolution for Semantic Image Segmentation. in *Computer Vision – ECCV 2018* (eds. Ferrari, V., Hebert, M., Sminchisescu, C. & Weiss, Y.) 833–851 (Springer International Publishing, Cham, 2018).

[50] Xie, E. *et al*. SegFormer: Simple and Efficient Design for Semantic Segmentation with Transformers. in *Advances in Neural Information Processing Systems* vol. 34 12077–12090 (Curran Associates, Inc., 2021).

[51] Perera, S., Erzurumlu, Y., Gulati, D. & Yilmaz, A. MobileUNETR: A Lightweight End-to-End Hybrid Vision Transformer For Efficient Medical Image Segmentation. in *Computer Vision – ECCV 2024 Workshops* (eds. Del Bue, A., Canton, C., Pont-Tuset, J. & Tommasi, T.) 281–299 (Springer Nature Switzerland, Cham 2025).

[52] Fu, Z., Wang, F., Ma, H., You, Q. & Feng, Y. Records of shallow landslides triggered by extreme rainfall in July 2024 in Zixing, China. *Figshare*10.6084/m9.figshare.27960762 (2025). 10.1038/s41597-025-05670-w PMC12325695 40764332

[53] Lu, W., Hu, Y., Zhang, Z. & Cao, W. A dual-encoder U-Net for landslide detection using Sentinel-2 and DEM data. *Landslides* 20, 1975–1987 (2023).

[54] Sameen, M. I. & Pradhan, B. Landslide Detection Using Residual Networks and the Fusion of Spectral and Topographic Information. *IEEE Access* **7**, 114363–114373 (2019).

[55] Tang, X. *et al*. FedLD: Federated Learning for Privacy-Preserving Collaborative Landslide Detection. *IEEE Geoscience and Remote Sensing Letters* **21**, 1–5 (2024).

